# Obstinate Intermittent Coryza—Instantaneous Cure

**Published:** 1852-08

**Authors:** A. Menudier


*Obstinate intermittent coryza—instantaneous cure.* By Dr. A. Menudier.—A lady, aged 24, had been for some years subject to severe attacks of coryza, coming on twice or three times in a week, and lasting from twelve to thirty-six hours. On one occasion, she was anxious to have the disease arrested immediately. M. Menudier thereupon applied a sinapism from the scapulæ to the loins : in a quarter of an hour, the coryza began to diminish, and in three quarters of an hour the patient could no longer bear the mustard. The skin was reddened; but the coryza was cured; and had not returned at the end of three months.—*Ib.*

